# Reverse shoulder arthroplasty following a diagnosis of bilateral posterior shoulder dislocation secondary to electric shock: a case report and literature review

**DOI:** 10.3389/fsurg.2026.1786014

**Published:** 2026-04-15

**Authors:** Weijin Miao, Xiaofei Liu, Min Wang, Haiquan Zeng, Xianmao Liu, Shaohua Liang, Wen Wang

**Affiliations:** 1 Guangzhou Red Cross Hospital, Jinan University, Guangzhou, Guangdong, China; 2 Jinan University, Guangzhou, Guangdong, China; 3 Ruyuan Yao Autonomous County People’s Hospital, Shaoguan, Guangdong, China; 4 Hui Ya Hospital of The First Affiliated Hospital, Sun Yat-sen University, Huizhou, Guangdong, China

**Keywords:** bilateral shoulder dislocation, case report, electric shock, posterior shoulder dislocation, reverse shoulder arthroplasty

## Abstract

A 62-year-old man presented with bilateral posterior shoulder dislocations 2 months after sustaining an accidental electric shock while fishing. Imaging confirmed chronic locked posterior dislocations with reverse Hill-Sachs lesions and large humeral head articular defects (approximately 60% on the left and 55% on the right), together with partial-thickness supraspinatus and subscapularis tendon tears. The patient underwent staged reverse total shoulder arthroplasty. At the 12-month follow-up, he was free from pain with a markedly improved active range of motion (forward flexion, 130°; abduction, 100°) and excellent functional outcomes (constant score: 86, left shoulder; 83, right shoulder; ASES, 90). This case supports reverse shoulder arthroplasty as a viable option for a chronic bilateral posterior shoulder dislocation with substantial humeral head defects.

## Introduction

Posterior shoulder dislocation is a rare injury, accounting for 1% to 4.7% of all shoulder dislocations (1). Bilateral posterior shoulder dislocations are even rarer, often resulting from seizures, trauma, or electric shock (2, 3). Electric shock leading to bilateral posterior shoulder dislocation is particularly uncommon, with fewer than 5% of cases attributed to this cause (1, 2, 4–6). This report describes the case of a 62-year-old man who sustained bilateral posterior shoulder dislocations following an electric shock who was successfully treated with staged reverse shoulder arthroplasty.

## Case history

A 62-year-old man presented with bilateral shoulder pain and restricted upper-limb function 2 months after an accidental electric shock while fishing. He suffered symptoms immediately after the incident; however, he denied experiencing numbness or paresthesia. Initially, he received analgesics and supportive care at an outside hospital. Because his pain progressively worsened, he sought further evaluation at our institution.

**Figure 1:**
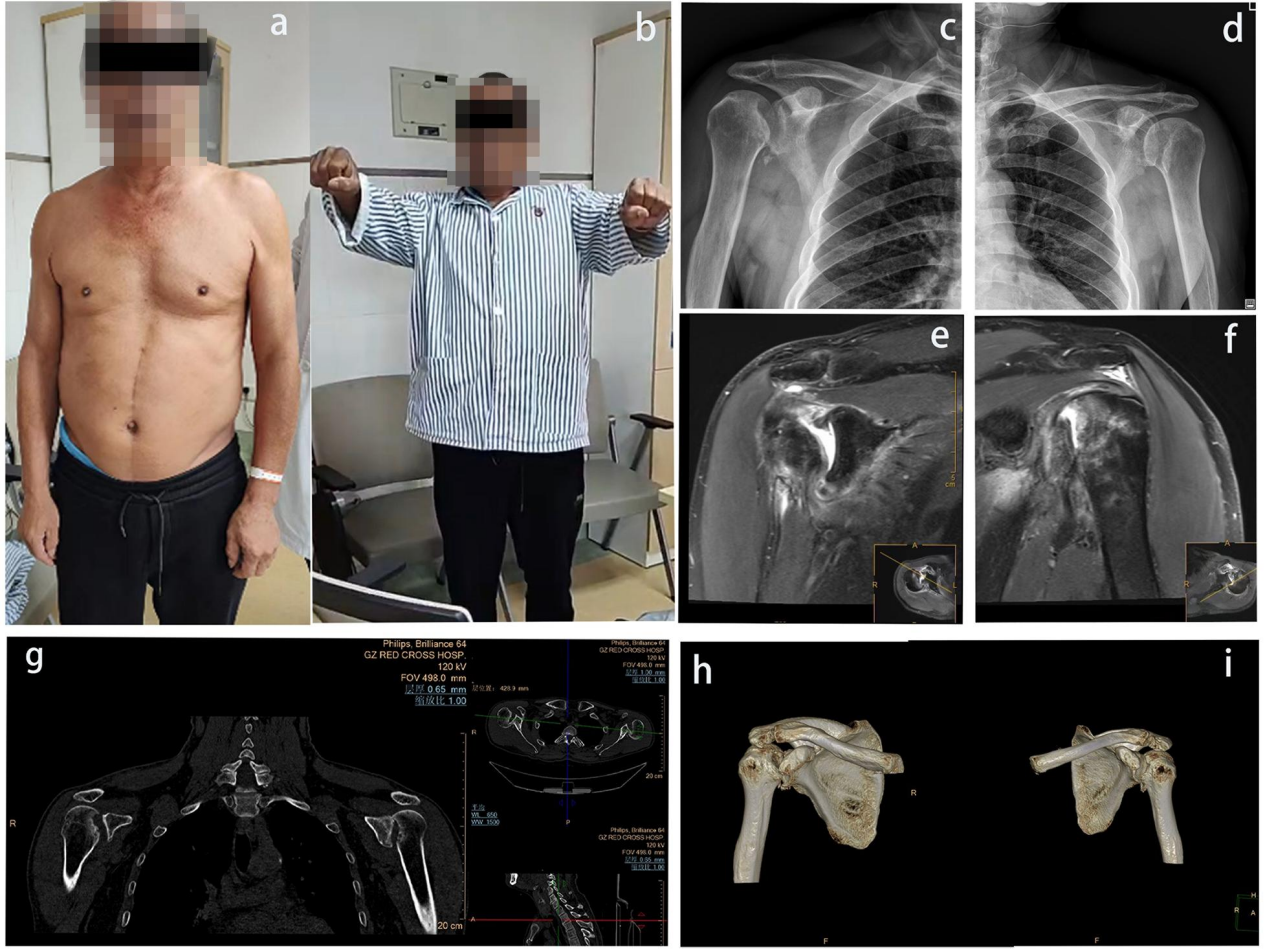
**(a,b)** A preoperative examination showing a limited shoulder range of motion; **(c,d)** preoperative anteroposterior (AP) radiographs; **(e,f)** an MRI demonstrating partial-thickness supraspinatus and subscapularis tendon tears with long head of the biceps tendon–superior labrum (SLAP complex) abnormalities; and **(g–i)** CT images showing a locked posterior dislocation of both shoulders with humeral head defects.

A physical examination revealed symmetrical, bilateral limitations in the range of motion (ROM) of both shoulders: flexion, 0°–80°; extension, 0°–5°; adduction, 0°–10°; abduction, 0°–80°; internal rotation, 0°–25°; and external rotation, 0°–20°. Posterior tenderness was present, and the results of both the painful arc and lift-off tests were positive. The Dugas sign was recorded but considered non-specific because it is primarily used for anterior dislocation and was not relied upon for diagnosing posterior dislocation. Muscle strength and sensation were preserved in both upper limbs (Figures 1a,b).

A radiographic analysis revealed chronic posterior dislocations of both shoulders (Figures 1c,d). Computed tomography (CT) (Figures 1g–i) confirmed chronic posterior dislocations with reverse Hill-Sachs lesions. Magnetic resonance imaging (MRI) (Figures 1e,f) revealed partial-thickness tears in the supraspinatus and subscapularis tendons, with abnormalities of the long head of the biceps tendon and the superior labrum (SLAP complex) in both shoulders. Electrocardiography (ECG) revealed atrial fibrillation.

Given the chronic locked posterior dislocations, the large humeral head articular defects (approximately 60% on the left with engagement on the posterior glenoid rim), the associated impaction and bony fragments, and the rotator cuff compromise on MRI, reverse shoulder arthroplasty (RSA) was considered the most reliable option to restore function. Considering the chronicity, atrial fibrillation, and more severe pain on the left side, a staged approach was planned after obtaining informed consent, with the left shoulder (larger defect) addressed first.

**Figure 2:**
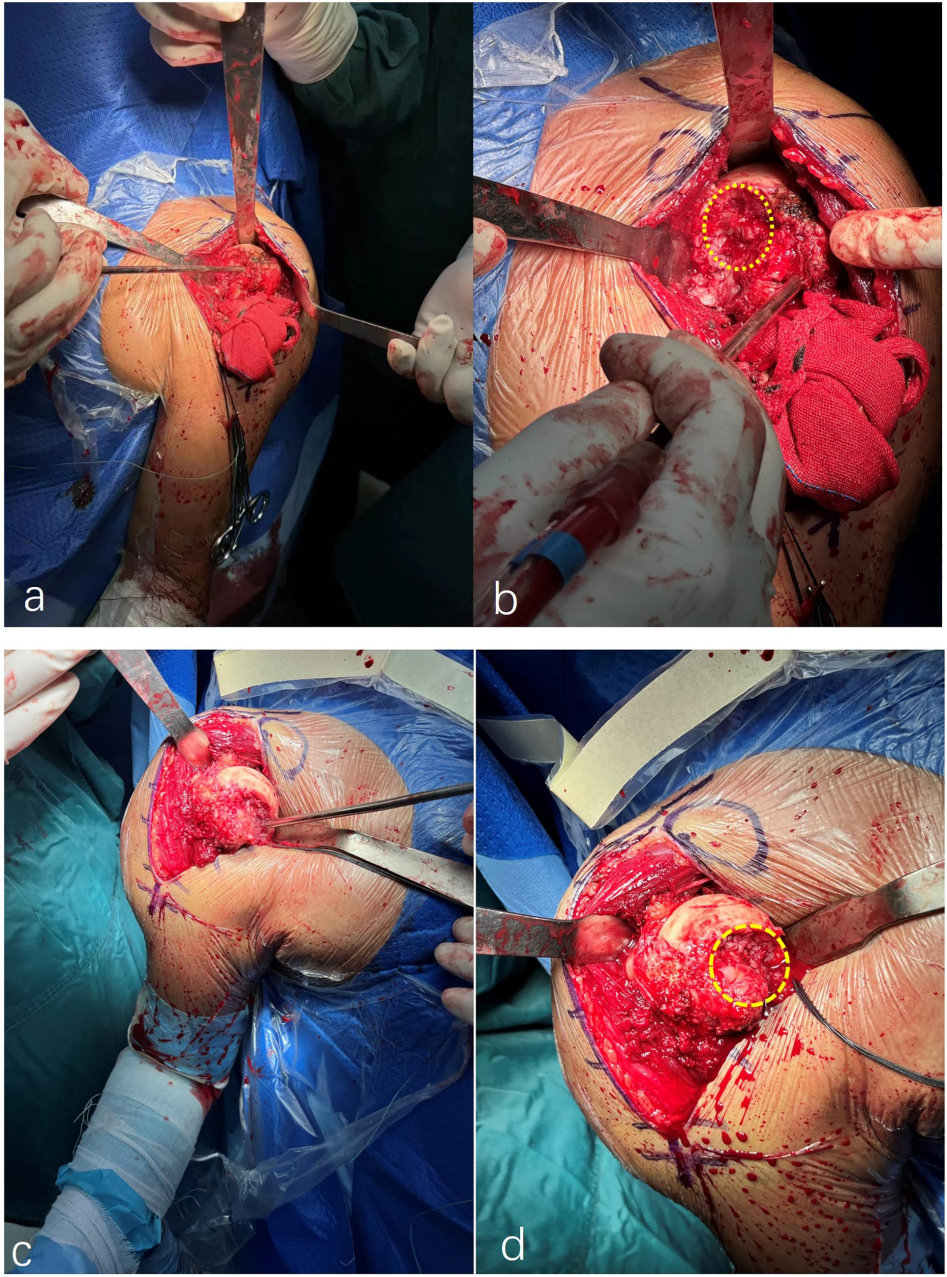
**(a,b)** Intraoperative findings showing a left humeral head defect of approximately 60% (yellow circle), and **(c,d)** a right humeral head defect of approximately 55% (yellow circle).

Surgery was performed in the beach chair position using a deltopectoral approach. After capsulotomy, dense fibrosis was observed. The humeral head was exposed, confirming posterior dislocation and an approximately 60% articular surface defect (Figures 2a,b). To facilitate exposure, the supraspinatus and infraspinatus tendons were carefully elevated, and the soft tissues around the glenoid were released to improve glenoid access. All implants were procured from the SMR Reverse Shoulder System (LimaCorporate S.p.A., Villanova, San Daniele, Friuli, Italy). A cementless S baseplate, 36-mm rotating platform humeral component, size-20 cementless humeral stem, 42-mm glenosphere, and  + 6-mm retentive (anti-dislocation) liner were implanted. The glenoid baseplate was fixed using screws. Larger glenospheres may increase jump distance and improve soft-tissue tensioning, thereby reducing postoperative instability (7, 8). The joint was reduced with appropriate tension, showing a full ROM without impingement or instability. The subscapularis was repaired, and the rotator cuff was reattached to the greater tuberosity. Subscapularis repair has been reported to improve American Shoulder and Elbow Surgeons (ASES) scores after RSA (9). The wound was irrigated with saline and then closed using non-absorbable sutures.

One month later, the right shoulder was addressed using the same technique, revealing a 55% humeral head defect (Figures 2c,d). The same implant system was used (SMR Reverse Shoulder System; LimaCorporate, Villanova, San Daniele Friuli, Italy): a cementless S baseplate, 36-mm rotating platform humeral component, size-21 cementless humeral stem, and 0-mm retentive (antidislocation) liner. Postoperative rehabilitation followed an evidence-informed arthroplasty rehabilitation framework: an abduction pillow was used for 6 weeks, with a neutral-rotation sling to protect the subscapularis repair. Active ROM exercises were initiated at the wrist and elbow, progressing to passive stretching using the Jackins method (10), and active elevation was introduced at 12 weeks (11).

**Figure 3:**
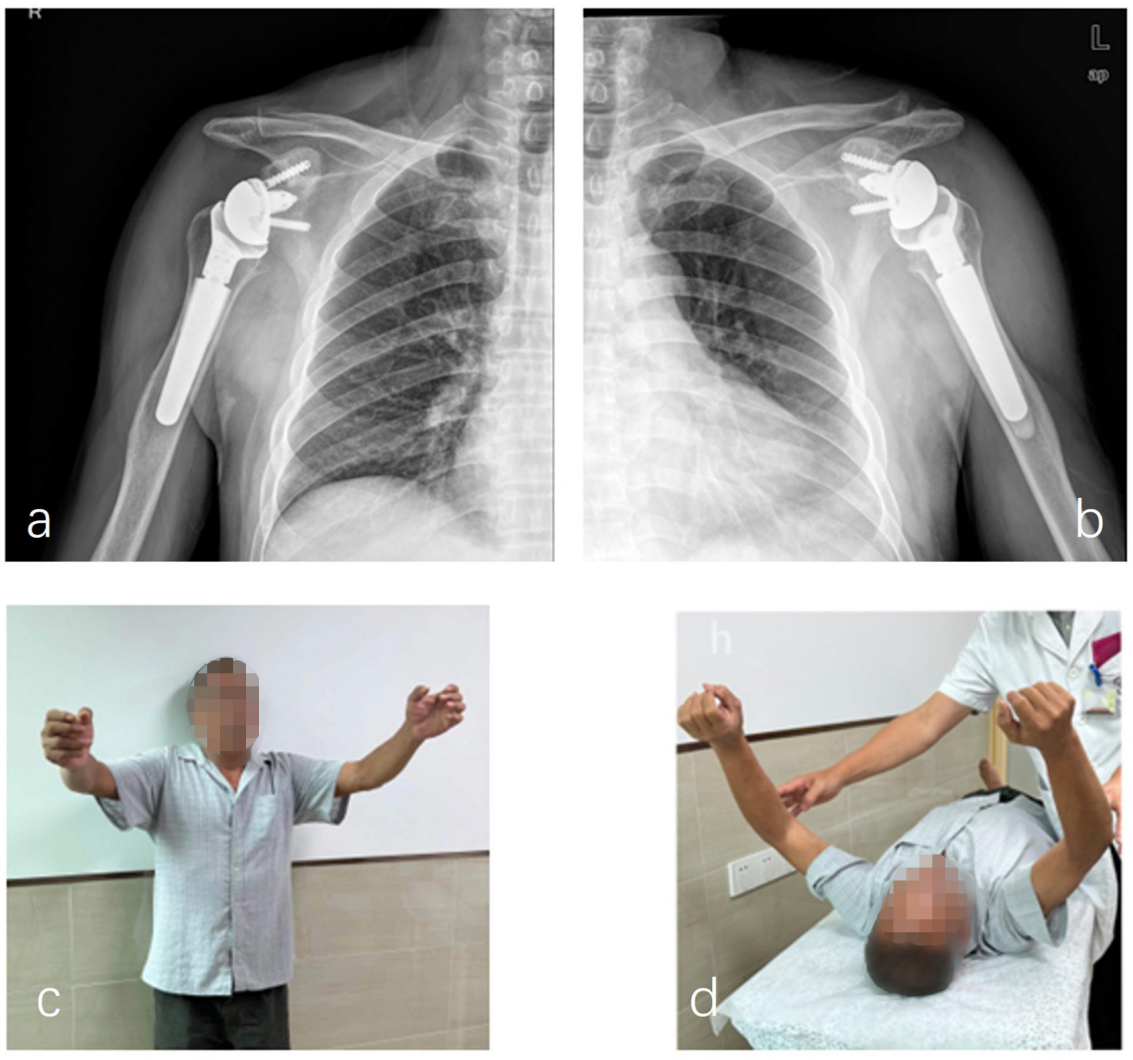
**(a,b)** A postoperative assessment of shoulder function in the clinic at 3 months. **(c,d)** Postoperative anteroposterior (AP) radiographs of both shoulders demonstrating prosthesis stability.

At 3 months after surgery, the left shoulder ROM was forward flexion 110°, abduction 90°, external rotation 45°, and internal rotation to L5, and home-based rehabilitation was sufficient without formal physiotherapy (Figures 3a,b). The right shoulder ROM was forward elevation 100°, abduction 90°, external rotation 40°, and internal rotation to L5. Formal physiotherapy was not required. At the 6-month follow-up, the patient was free from pain, and imaging confirmed osseous healing without any abnormal findings (Figures 3c,d). At 12 months, the patient demonstrated an excellent ROM (forward flexion, 130°; abduction, 100°; and internal rotation to L4) (Supplementary Video S1). Functional outcomes were excellent (constant score: 86, left shoulder; 83, right shoulder; ASES, 90). The patient reported no pain, a sufficient ROM for activities of daily living, and overall satisfaction with the outcome.

## Discussion

To clarify the literature context, we performed a focused narrative review of (1) a bilateral posterior shoulder dislocation after electric shock and (2) arthroplasty-based treatment for chronic/locked posterior dislocation. PubMed and Google Scholar were searched from inception to 1 February 2026, using combinations of “posterior shoulder dislocation,” “bilateral,” “electric shock”/“electrical injury”/“electrocution,” “locked,” “reverse shoulder arthroplasty,” “hemiarthroplasty,” and “shoulder arthroplasty.” We included adult case reports and clinically relevant reviews addressing electrical injury–related bilateral posterior dislocation and operative strategies for chronic/locked cases with substantial humeral head defects. We excluded non-shoulder injuries, purely anterior instability, pediatric-only reports, and abstracts without full texts. The reference lists of the included papers were also screened to identify additional relevant publications. The key referenced studies and their main findings are summarized in Table 1. The search was reported in line with PRISMA-S principles, and the narrative synthesis was structured according to published guidelines for narrative reviews (12–14).

**Table 1:** Summary of key referenced studies and their main findings.

Ref	Study type/setting	Etiology/population	Chronicity and key pathology	Treatment	Main findings relevant to the current case
(1)	Case report	Bilateral posterior fracture dislocation after electrical shock	Acute/unspecified; fracture dislocation	Bilateral hemiarthroplasty	Reports bilateral posterior fracture dislocation after electric shock treated with bilateral hemiarthroplasty, illustrating that arthroplasty may be required in severe electrical injury cases and that diagnosis can be delayed.
(6)	Two case reports + literature review	Bilateral posterior fracture dislocation	Variable; fracture dislocation	Operative (varied)	Presents two cases and a brief review, emphasizing that posterior fracture dislocation can follow seizures, trauma, or electrical injury, and that treatment should be tailored to chronicity and defect size.
(15)	Review	Locked posterior shoulder dislocation	Chronic/locked; defect size critical	Algorithm, including McLaughlin, grafting, and arthroplasty	Outlines a practical approach to locked posterior dislocation; larger defects and longer-standing dislocations generally shift management toward arthroplasty rather than reconstruction.
(16)	Systematic review	Posterior shoulder fracture dislocation	Acute and chronic	Multiple surgical options	Synthesizes available surgical options for posterior fracture dislocation and reinforces that substantial humeral head defects (often ∼50% or more) and chronicity are key drivers when considering arthroplasty.
(17)	Review/clinical series review	Reverse total shoulder arthroplasty by etiology	Multiple etiologies include cuff tear arthropathy, etc.	RSA	Summarizes outcomes of reverse shoulder arthroplasty across indications, supporting its reliability for pain relief and elevation in cuff-deficient shoulders, with results influenced by the underlying etiology.
(18)	Registry/large cohort analysis	RSA survivorship by indication, age, and gender	Long-term survivorship	RSA	Registry-based evidence shows durable implant survivorship across indications, supporting RSA as a reasonable long-term option in appropriately selected patients.

Bilateral posterior shoulder dislocations are rare and often misdiagnosed, with electric shock representing a particularly uncommon etiology (19). Early diagnosis is essential to prevent chronic dysfunction (20). Acute cases are typically managed with closed reduction. In chronic presentations, such as this patient, who presented 2 months postinjury with significant joint damage, surgical intervention is often required. Treatment options include open reduction and internal fixation (ORIF), the McLaughlin or modified McLaughlin procedure, allograft or autograft humeral head reconstruction, anatomic shoulder arthroplasty, or reverse shoulder arthroplasty (RSA) (15, 16). Selection depends on dislocation chronicity, humeral head defect size, glenoid integrity, and patient factors such as age, occupation, and activity level (1, 6, 21).

In this patient, a preoperative CT and MRI revealed significant structural damage, indicating that achieving stable reduction would be challenging (22). For patients with massive irreparable rotator cuff tears and humeral head defects exceeding 50%, traditional joint-preserving procedures may be inadequate (23). RSA is generally considered for patients with preserved deltoid function, irreparable rotator cuff deficiency, large humeral head defects (>50%), and chronic irreducible dislocation (17, 24). In our patient, an MRI demonstrated supraspinatus and subscapularis partial-thickness tears, supporting rotator cuff compromise; therefore, RSA was chosen to address the humeral head defects and restore mobility (18). A distinctive feature of this patient was the occurrence of atrial fibrillation after an electric shock. A nationwide cohort study reported no increased long-term mortality among immediate survivors of accidental electric shock but identified cardiac complications in a subset of patients (25). In the available literature on electric shock–related bilateral posterior shoulder dislocation, reported treatments have included hemiarthroplasty or other reconstructive procedures; we did not identify any previous reports in which both shoulders were treated with RSA for this etiology (1, 4, 5, 26). Delayed arrhythmias have been reported after electric shock, which has been associated with heart failure, cardiomyopathy, and myocardial infarction (27). Most clinically relevant arrhythmias in patients presenting after an electric shock can be detected by performing an admission ECG (28). The patient's atrial fibrillation complicated the perioperative management and supported a staged surgical approach.

Delayed diagnosis elevates the risk of complications, including failure of closed reduction, shoulder stiffness, avascular necrosis, humeral head collapse, post-traumatic arthritis, and muscle atrophy, complicating surgical management (20). In the initial procedure (left shoulder), the locked posterior dislocation posed challenges because of limited exposure and difficult reduction. Intraoperative reduction was achieved, but it was unstable, with recurrent dislocation. A spider arm retractor was used to maintain the arm position and apply proximal force at the elbow, enabling sustained humeral head subluxation until prosthesis implantation was performed.

One month later, the right shoulder was addressed using the beach chair position and spider arm setup, informed by the first procedure. Although a 55% humeral head defect was identified, reduction and prosthesis placement were less challenging. Rehabilitation was implemented as a staged, protected-to-progressive loading program to balance stability (including protection of the repaired subscapularis) and functional recovery, consistent with published arthroplasty rehabilitation consensus principles and the broader literature noting variability across postarthroplasty protocols (11).

At the 12-month follow-up, the patient reported satisfaction with both pain relief and functional recovery, noting no complications (Supplementary Video S1).

### Summary

Reverse shoulder arthroplasty is a viable treatment option for older patients with chronic bilateral posterior shoulder dislocations secondary to electric shock, especially when associated with large humeral head defects and rotator cuff damage. The case of the patient in this study demonstrates successful outcomes with significant pain relief and functional improvement 12 months postoperatively.

## Data Availability

The original contributions presented in the study are included in the article/Supplementary Material, and further inquiries can be directed to the corresponding author/s.

## Supplementary material

Supplementary Video 1 A postoperative video showing that the patient reported no pain, had an adequate ROM for activities of daily living, and was satisfied with the outcome.
